# Comparative assessment of dental care services utilization and barriers among individuals with and without intellectual and developmental disabilities in Jordan

**DOI:** 10.7717/peerj.21447

**Published:** 2026-06-18

**Authors:** Sabha Mahmoud Alshatrat, Wael Mousa Al-Omari, Abedelmalek Kalefh Tabnjh, Majd Alsaleh, Hasan Subhi

**Affiliations:** 1 Department of Applied Dental Sciences, Faculty of Applied Medical Sciences, Jordan University of Science and Technology, Irbid, Jordan; 2 Department of Prosthodontics, Faculty of Dentistry, Jordan University of Science and Technology, Irbid, Jordan; 3 Department of Cariology, Odontology School, University of Gothenburg, Gothenburg, Sweden; 4 Department of Pathology, Saveetha Medical College and Hospital, Saveetha Institute of Medical and Technical Sciences, Chennai, Tamil Nadu, India; 5 Department of Pediatric Dentistry, College of Dentistry, University of Illinois Chicago, Chicago, Illinois, USA; 6 College of Dentistry, Al-Furqan University, Mosul, Iraq

**Keywords:** Access to dental care, Barriers to dental care, Intellectual and developmental disabilities, Jordan

## Abstract

**Background:**

Oral health is a key component of overall well-being, yet individuals with intellectual and developmental disabilities (IDD) often face significant barriers to accessing proper dental care. In Jordan, limited research has examined these disparities, underscoring the need for a focused investigation.

**Objective:**

This study aimed to assess dental service utilization among individuals with IDD in Jordan and identify key barriers to dental care access compared with individuals without IDD.

**Methods:**

A cross-sectional comparative study was conducted with 317 participants: 168 with IDD and 149 without. Data were collected using a validated, self-designed, closed-ended questionnaire that assessed dental service utilization, reasons for dental visits, and perceived barriers to care. Written informed consent was obtained from all participants or caregivers before participation. Participants with IDD were recruited from registered IDD centers, while individuals without IDD were recruited from the same geographic areas. Convenience sampling was used to select participants from centers affiliated with IDD. Data were analyzed using SPSS version 27, with a significance level set at *P* < 0.05. Group differences were evaluated with Chi-square tests and contingency tables.

**Results:**

Reported barriers included long waiting times, high treatment costs, inconvenient clinic hours, feelings of embarrassment, lack of specialized dental staff, limited provider knowledge on treating individuals with disabilities, and inadequate facilities. No significant differences were found between the two groups on lack of dental insurance or dental care access anxiety.

**Conclusions:**

This study confirms socioeconomic disparities in dental service utilization among individuals with IDD in Jordan and highlights multiple barriers to dental care access. These findings identify the most perceived obstacles to oral health services among individuals with IDD, offering valuable insights for developing targeted health policies to improve access to dental care and reduce oral health inequalities in this vulnerable population.

## Introduction

Oral health is a vital part of overall health, significantly affecting physical, social, and psychological well-being (Kane, 2017). Good oral hygiene helps prevent dental issues such as tooth decay, gum disease, and oral infections, which can significantly affect a person’s quality of life (Löe, 2000). However, maintaining good oral health can be particularly challenging for individuals with intellectual and developmental disabilities (IDD) (Wilson et al., 2019b).

Intellectual disability (ID) is characterized by limitations in intellectual functioning and adaptive behavior (Fisher, 2012; Schalock, Luckasson & Shogren, 2007; Schalock, Luckasson & Tassé, 2010) and is considered a subset of IDD. It is commonly associated with developmental disabilities, defined as impairments in cognitive functioning with onset during the developmental period, typically before the age of 22 (Fisher, 2012; Prater & Zylstra, 2006). As a result, individuals with IDD may face challenges across multiple domains, including self-care, communication, learning, mobility, independent living, and economic self-sufficiency (Fisher, 2012; Prater & Zylstra, 2006). These difficulties, combined with reliance on others for daily activities, barriers to accessing health care, and lower socioeconomic and educational status, contribute to disparities in health outcomes among this population (Wilson et al., 2019b; Braun, Yeargin-Allsopp & Lollar, 2009; Alshammari, Doody & Richardson, 2018; Slack-Smith, Ree & Leonard, 2010).

Oral health disparities are particularly evident among individuals with IDD, who are more susceptible to poor oral hygiene and complex care needs (Morgan et al., 2012). Compared with the general population, they have a higher prevalence and severity of periodontal disease and untreated caries (Anders & Davis, 2010). Furthermore, poor oral health not only compromises daily functioning but also contributes to systemic conditions such as cardiovascular disease, respiratory disease, stroke, aspiration pneumonia, and diabetes mellitus (Aida et al., 2011; Joshipura et al., 2003; Tada & Miura, 2012; Lalla & Papapanou, 2011). In addition to these medical concerns, pain, aesthetic concerns, nutritional limitations, and functional difficulties can adversely affect their psychological and social well-being (Spanemberg et al., 2019).

Implementing effective interventions to improve oral health care for individuals with IDD requires a comprehensive understanding of the barriers they face. Global evidence consistently shows poor oral health and inadequate access to care among this population. In Australia, 41% of children with IDD needed simple treatment, yet many needs went unmet (Desai, Messer & Calache, 2001). Similar trends are reported worldwide: high levels of caries, gingivitis, and tooth loss in Serbia (Petrović et al., 2016); poor-quality dental care for Dutch children with severe disabilities (De Jongh et al., 2008); and a 49.5% prevalence of untreated caries among Brazilian children with cerebral palsy (De Camargo & Antunes, 2008). In Germany, 89.4% of adults with intellectual disability had caries and more missing teeth than the general population (Schmidt et al., 2021). A systematic review from India reported pooled prevalence rates of 64% for caries, 38% for poor oral hygiene, and 54.2% for periodontal disease (Mehta et al., 2024). Studies in Ethiopia (Tefera et al., 2022) and Lebanon (Diab et al., 2017) further confirm high rates of dental caries and poor oral health among individuals with IDD.

In Jordan, limited studies have focused on individuals with autism (Alshatrat, Al-Bakri & Al-Omari, 2020) and Down syndrome (Habashneh et al., 2012), and there is a notable lack of research addressing the broader population of individuals with IDD and their access to routine oral health services (Alshatrat et al., 2024). This study focuses on the broader population of individuals with IDD for several reasons. First, individuals with different forms of IDD often face common systemic barriers to accessing dental care, including limited availability of trained providers, transportation challenges, financial constraints, and insufficient disability-friendly dental services. These barriers are largely not diagnosis-specific but reflect broader structural issues within the healthcare and dental systems. Furthermore, policymakers and service planners in Jordan typically design oral health services for the overall IDD population rather than for isolated diagnostic subgroups. Therefore, understanding barriers at the population level provides evidence to support the development of inclusive policies, subgroup-specific training programs, and service delivery models that benefit individuals across the IDD spectrum. In addition, this study provides an important baseline for future research. The findings can guide more targeted investigations into the unique needs of subgroups within the broader IDD population. Accordingly, this study aims to identify barriers to accessing oral health care among individuals with IDD in Jordan, compared with their peers without IDD.

## Materials and Methods

This study was conducted in full accordance with the World Medical Association Declaration of Helsinki, and the study protocol was approved by the Institutional Review Board of Jordan University of Science and Technology (Reference: 2017/0032). A cross-sectional survey was conducted to identify barriers to accessing oral healthcare among individuals with intellectual and developmental disabilities (IDD) in Jordan.

The study was conducted across multiple regions of Jordan, including the northern, central, and southern areas, and recruited participants from registered intellectual and developmental disability (IDD) centers and community locations. Jordan has a diverse socio-economic population, with variations between urban and rural areas. The formal and predominant language is Arabic, which guided the development of a self-designed Arabic questionnaire to ensure cultural and linguistic relevance for all participants. Content validity was assessed by two expert panels, yielding an average congruency percentage (ACP) of 92%, indicating high agreement regarding item relevance and appropriateness. Reliability was evaluated using test–retest administration with 10 caregivers of individuals with IDD, yielding a Cronbach’s alpha coefficient of 0.75, indicating acceptable internal consistency. A pilot study with 10 caregivers of individuals with IDD (not included in the final sample) to assess clarity, content, and format, and feedback was incorporated into the final questionnaire. The final questionnaire included three sections: (1) Demographic information (five items) of participants and caregivers; (2) Barriers to dental care (13 items), adapted and modified from the literature (AlShehri, 2012; Knoer & Sigal, 2009; Prabhu et al., 2010); (3) Dental service utilization (five items).

A list of recognized intellectual and developmental disability (IDD) centers was obtained from the Jordanian Ministry of Social Development. A convenience sampling method was employed to recruit participants from centers located in the northern, southern, and central regions of Jordan, based on the willingness of center managers to facilitate the distribution.

The severity of disability for participants with IDD was obtained from official records provided by the center managers, based on professional diagnostic evaluations documented in participants’ files, rather than self-reports. These data were collected for descriptive purposes only and were not included as analytical variables in the statistical comparisons.

In the participating centers, the paper-based questionnaire was distributed to parents or legal caregivers, who completed it on behalf of individuals with IDD. To ensure ethical compliance, a written informed consent form was provided with the questionnaire. Participants were instructed to read, sign, and return the form if they agreed to participate, in accordance with the IRB-approved protocol. The consent form explicitly informed participants of their right to withdraw from the study at any time without justification, explained what would happen if they chose to stop participating, and provided contact information for questions or concerns. This dual approach—using a detailed written consent form and the cover letter—was adopted to maximize participation while maintaining ethical standards. For the comparison group, individuals without IDD were recruited from the same geographic areas (*e.g.*, schools, parks, malls) using convenience sampling, and the same consent process was applied.

Questionnaires were collected within 1 to 3 weeks of distribution. For non-respondents at the centers, a reminder note was sent after three weeks to encourage participation. Data collection was conducted between July 2021 and January 2022.

Sample size was calculated using G*Power software, based on Alshatrat, Al-Bakri & Al-Omari (2020), with 80% power and a 5% margin of error. The minimum required sample size was 133 participants per group. A total of 200 questionnaires were distributed in each group. Of these, 168 were returned by caregivers of individuals with IDD and 149 by individuals without IDD.

### Statistical analysis

Data were analyzed using IBM SPSS^^®^^ version 27 (IBM-SPSS, Armonk, NY, USA) with the assistance of a biostatistician. Descriptive statistics were calculated for continuous (mean, SD) and categorical variables (frequency, percentage). Associations were tested using Pearson’s Chi-square, with significance set at *p* < 0.05.

## Results

Of 200 eligible participants in each group, 168 individuals with IDD (84%) and 149 without IDD (75%) completed the questionnaire, yielding response rates of 84% and 75%, respectively. Ages ranged from 8 to 59 years (mean: 30 years) in the IDD group and from 7 to 75 years (mean: 23 years) in the non-IDD group. The male-to-female ratio was comparable between the group with IDD (61%: 39%) and the group without IDD (67%: 32%). Moreover, 90% of people in both groups were single, and there was a significant difference in average educational level between the two groups, with individuals without IDD having a higher level (*P* < 0.05). Other demographic details are shown in Table 1, including the location of developmental delay centers, the level of disability among participants with IDD, insurance coverage, and family income.

**Table 1:** Comparison of sociodemographic characteristics between participants with and without IDD.

**Characteristics**	**IDD**		**Without IDD**	
**Family income (Jordanian Dinar)**	***n***	**(%)**	***n***	**(%)**
<250	97	(57.7%)	2	(1.3%)
250–500	68	(40.5%)	70	(47.0%)
500–1,000	1	(0.6%)	64	(43.0%)
>1.000	2	(1.2%)	13	(8.7%)
**Age**				
< 18	32	(19%)	97	(65%)
18–40	95	(56.5%)	37	(25%)
>40	41	(24.4%)	15	(10%)
**Insurance**				
Yes	138	(82.1%)	100	(67.1%)
No	29	(17.3%)	49	(32.9%)
**Disability center/region**				
Amman	41	(24.4%)	55	(36.9%)
North	113	(67.3%)	68	(45.6%)
South	14	(8.3%)	26	(17.4%)
**Severity of disability**				
None	0	(0%)	149	(100%)
Mild	19	(11.3%)	0	(0%)
Moderate	57	(33.9%)	0	(0%)
Severe	92	(54.8%)	0	(0%)

Compared with individuals without IDD, those with IDD did not differ significantly in the time since their last dental appointment. The majority had seen a dentist within the past year (64% *vs.* 57%, respectively) (*P* value > 0.05).

**Figure 1:**
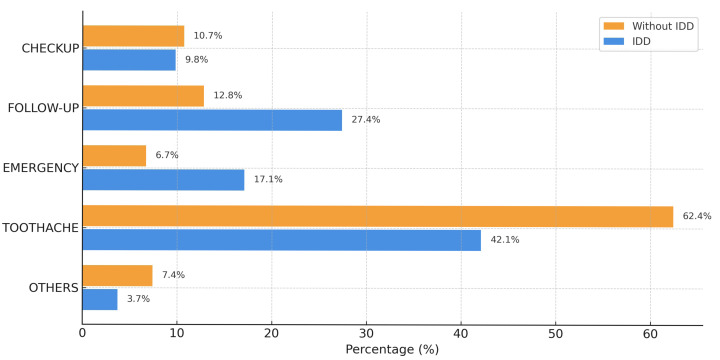
Reasons for last visit to dental service for individual with and without IDD.

Among individuals with IDD, 42.1% reported that treatment for a toothache was the primary reason for their most recent dental visit, compared with 62.4% of individuals without IDD. This difference was statistically significant (*P* < .001). In contrast, routine checkups were the least frequently cited reason for a recent dental visit among individuals with developmental delay, reported by only 9.8% (Fig. 1).

Using a chi-square test, Table 2 presents the common barriers people encounter when trying to access and use dental services. There was a significant difference in the likelihood of reporting most barriers, with those with IDD significantly more likely to report them than individuals without IDD (*P* value <0.01). These barriers included lengthy wait times, high costs, inconvenient dental clinic hours, embarrassment, a lack of specialized dental staff, a lack of knowledge about how to treat people with disabilities, and inadequate facilities. However, there was no significant difference between the two groups regarding lack of insurance and dental anxiety (*P* value > 0.05).

**Table 2:** Comparison of reported barriers to dental care between individuals with and without IDD.

**Barrier**	**IDD** ***n*** ** (%)**	**Without IDD** ***n*** ** (%)**	***P*** ** value**
Can not afford the cost	95 (56.5)	57 (38.3)	0.003^*^
Dental office is too far away	75 (44.6)	19 (12.8)	0.000^*^
Dental office is not open at convenient times	71 (42.3)	40 (26.8)	0.004^*^
Dental office has no or difficult access for wheelchairs	102 (60.7)	10 (6.7)	0.000^*^
Dental office has inaccessible parking areas	83 (49.4)	9 (6.0)	0.000^*^
Dental office has a small space	63 (37.5)	7 (4.8)	0.000^*^
Dental office has inadequate facilities to provide dental care	91 (54.2)	5 (3.4)	0.000^*^
Dentist’s lack of knowledge of how to treat people with disability	78 (46.4)	9 (6.1)	0.000^*^
Dental office has a general dentist, not a specialist	57 (33.9)	14 (9.4)	0.000^*^
Long waiting time	103 (61.3)	62 (41.6)	0.000^*^
Fear of dental work	85 (50.6)	77 (51.7)	0.847
No insurance coverage/dental coverage	63 (37.5)	52 (34.9)	0.631
Embarrassment or any psychological barriers	73 (43.5)	14 (9.4)	0.000^*^

**Notes.**

* Significant difference, *p* < 0.05.

## Discussion

IDD are conditions common in pediatric clinics (Mithyantha et al., 2017) and are often associated with delays in social, physical, linguistic, and cognitive development. Most cases are attributed to genetic causes rather than metabolic disorders (Masri et al., 2023). Early diagnosis is critical for timely and effective interventions (Moeschler & Shevell, 2014).

It is well established that individuals with IDD have poorer oral health than the general population, with significant consequences for overall health (Wilson et al., 2019a; Hsieh et al., 2018). This disparity highlights the extent of oral health inequity and exposes gaps in current healthcare systems (Ward et al., 2019). Strategies such as improving access, enhancing caregiver support, and promoting oral health education are essential to addressing these inequities.

To the best of our knowledge, no prior studies have examined barriers to oral health services among Jordanian individuals with IDD. Therefore, this study provides an important contribution by identifying these barriers and informing strategies to improve accessibility.

Response rates differed across groups: 84% among participants with IDD and 75% among individuals without IDD, yielding an average of 79%. Several factors may explain this difference. First, participants with IDD were recruited through structured centers, and caregivers completed the questionnaires, which likely increased participation. In contrast, most of the comparison group was under 18 years old and may have been less aware of the study’s relevance or importance, potentially contributing to the lower response rate. Additionally, recruiting at community locations may have imposed time constraints or reduced motivation to participate. Although the difference is relatively small, it may introduce a minor participation bias that should be considered when interpreting the findings. Findings showed that toothache was the most common reason for dental visits in both groups. However, individuals without IDD were significantly more likely to visit the dentist only when in pain. This pattern suggests symptomatic rather than preventive care, often leading to delayed visits until oral conditions progress to urgent, severe dental problems. Compliance with follow-up appointments was higher among individuals with IDD, likely reflecting the need for continued care after urgent treatment. This may reflect strong caregiver awareness of the importance of the oral health of individuals with IDD in their care. The lack of self-autonomy among individuals with IDD over their health may have intensified caregivers’ sense of responsibility to attend follow-up appointments. Although not examined in the present study, the observed high rate of post-treatment complications may have contributed to increased compliance with follow-up appointments. This may be particularly relevant for individuals with IDD, who are often presumed to have greater difficulty adhering to post-operative instructions, potentially leading to a higher risk of complications. However, patterns of follow-up attendance were not evaluated in this study and call for further investigation in future research.

Another possible explanation is that most individuals without IDD are under 18 years old. At this age, compliance with follow-up appointments is likely to be low.

In both groups, routine dental checkups were infrequent, despite strong evidence linking them to improved oral and overall health outcomes (World Health Organization, 2021; Iwasaki et al., 2021; Janto et al., 2023). Oral health education emphasizing preventive measures for oral diseases should be integrated into primary, secondary, and tertiary education programs and delivered through all available channels to reach the majority of the population.

Caregivers of individuals with IDD reported multiple barriers, including geographic, physical, behavioral, cultural, human resource, and financial challenges (World Health Organization, 2011; Bastani et al., 2021). These barriers may impede timely preventive care and worsen health disparities.

Insurance coverage was not perceived as a barrier in either group, showing that governmental health services provide a basic level of access. In Jordan, most of the population, especially children, the elderly, and individuals with mental or physical disabilities, has access to basic medical and dental services. However, financial limitations continued to be a significant obstacle for caregivers of individuals with IDD. Governmental services largely lack adequate facilities, necessary equipment, properly trained specialized dentists, and timely appointments to address the urgent needs of people with disabilities, including people with IDD. Therefore, private, uninsured dental services might be necessary. Because most participants with IDD came from low-income families, treatment costs were perceived as a significant barrier. This also shows that, despite insurance coverage, it is likely insufficient to cover the specialized dental care often required. Similar findings have been reported elsewhere, linking socioeconomic disparities to reduced dental service utilization and poorer oral health outcomes (Sarkar et al., 2017; Zickafoose, Smith & Dye, 2015; Alshahrani & Raheel, 2016; Kane et al., 2008; Kancherla, Van Naarden Braun & Yeargin-Allsopp, 2013; Kranz et al., 2020; Sahab et al., 2022).

Fear of dental treatment was reported by about half of the participants in both groups, with no significant between-group differences. This aligns with evidence that dental anxiety affects a significant proportion of the general population (Armfield, 2010). In individuals with IDD, anxiety may be influenced by age and disability severity, which could account for the absence of group differences observed in this study (Pruijssers et al., 2014; Fallea, Zuccarello & Calì, 2016). New behavior guidance techniques, such as audio or VR distraction, may be beneficial but remain underexplored in patients with IDD. (Mehrotra, Shetty & Rai, 2024).

Physical and logistical barriers were also prominent. Caregivers reported difficulties with wheelchair access, inadequate facilities, long wait times, limited or no parking, and inconvenient clinic hours. These obstacles align with earlier reports (Adyanthaya et al., 2017; Bahadori, Ravangard & Asghari, 2013) and underscore the need to design dental facilities that are inclusive and accessible.

Human resource limitations were observed, particularly a shortage of dentists trained to care for individuals with IDD. Caregivers expressed concerns about gaps in provider knowledge and the limited availability of specialists, consistent with earlier findings (Williams, Spangler & Yusaf, 2015). Cultural barriers, including embarrassment and stigma, also restrict access. These findings emphasize the need for increased provider training, caregiver support, and public awareness to promote inclusivity and reduce stigma. Although the literature on barriers to dental services for individuals with IDD is limited, the current results align with earlier research indicating that cost, accessibility, and limited specialized services are major obstacles (Khan, Md Sabri & Ahmad, 2022; Zare et al., 2024). Solutions to these barriers may include tailored oral health education, improved access pathways, caregiver empowerment, culturally inclusive programs, and policies that ensure affordable specialized care.

## Limitations

This study has several limitations. Although convenient and cost-effective, convenience sampling may compromise sample representativeness and limit the generalizability of the results to the broader population. While it includes a subset of the population with IDD and makes them easily accessible to researchers, the findings primarily represent caregiver perspectives rather than those of individuals with IDD. The sample includes only institutionalized individuals, which may not represent the broader population of individuals with IDD, including those who are not institutionalized. This may introduce selection bias. Although the severity of disability was obtained from official records, it was not analyzed in relation to barriers to healthcare use in this study. Examining severity as an analytical variable could offer meaningful insights into whether individuals with more severe disabilities meet greater challenges in accessing care. Future studies are needed to explore the relationship between disability severity and healthcare use patterns to better understand the factors influencing access to health services among individuals with IDD. There was also a mismatch in age and family income distributions between groups, which may affect the validity of the results. However, for this population, convenience sampling may be a practical approach.

Although a reliable questionnaire was used, direct interviews could have yielded more detailed data. Despite these limitations, to the best of our knowledge, this is the first study to examine barriers to dental services among individuals with IDD. As such, it offers valuable insights into a disadvantaged population and can serve as a baseline for larger, more comprehensive studies on oral health needs and barriers to care.

## Conclusions

This study highlights significant socioeconomic disparities in the use of dental services among individuals with intellectual and developmental disabilities (IDD) and identifies multiple barriers to care. The findings reveal common challenges faced by this population, including long wait times, high treatment costs, limited availability of specialized dental staff, and inadequate facilities. These results can guide policy development, enhance dental professional training programs, improve service delivery, and ultimately improve access to dental care and reduce oral health inequalities among this vulnerable population.

## Supplemental Information

10.7717/peerj.21447/supp-1 Supplemental Information 1 Dataset

10.7717/peerj.21447/supp-2 Supplemental Information 2 STROBE checklist

10.7717/peerj.21447/supp-3 Supplemental Information 3 English version of the questionnaire

10.7717/peerj.21447/supp-4 Supplemental Information 4 Arabic version of the questionnaire

## Additional Information and Declarations

### Data Availability

The following information was supplied regarding data availability: The data is available in the Supplemental Files.
