# Supplementary material for: Comparative assessment of dental care services utilization and barriers among individuals with and without intellectual and developmental disabilities in Jordan
Source: PeerJ. 2026 Jun 18;14:e21447. doi: 10.7717/peerj.21447 (PMC13283370; doi:10.7717/peerj.21447)
Supplement: Supplemental Information 2 [file peerj-14-21447-s002.doc]

STROBE Statement—Checklist of items that should be included in reports of ***cross-sectional studies***

|  | Item No | Recommendation |
| --- | --- | --- |
| **Title and abstract** | 1 | Indicate the study’s design with a commonly used term in the title or the abstract  “Comparative Assessment of Access and Barriers to Dental Care among Individuals with and without [Developmental Delays](https://search.proquest.com/openview/c09c6fd4f608e7f674264de321021068/1?pq-origsite=gscholar&cbl=18750&diss=y)  in Jordan”  **Page #1**  **Methods**: A cross-sectional comparative study was conducted among 317 participants, comprising 168 individuals with IDD and 149 individuals without IDD.  **Page # 2** |
|  |  | Provide in the abstract an informative and balanced summary of what was done and what was found  Objective: This study aimed to assess the utilization of dental services among individuals with IDD in Jordan and to identify key barriers affecting their access to dental care, compared to individuals without IDD. Methods: A cross-sectional comparative study was conducted among 317 participants, comprising 168 individuals with IDD and 149 individuals without IDD. A validated, self-designed closed-ended questionnaire designed to gather data on dental service usage, reasons for dental visits, and perceived barriers to accessing care. Convenience sampling was employed to recruit participants from centers associated with IDD. Data were analyzed using SPSS® version 22, with a significance level set at P < 0.05. Chi-square tests and contingency table analyses assessed group differences. Results: Reported barriers included lengthy wait times, high treatment costs, inconvenient clinic hours, embarrassment, lack of specialized dental staff, limited provider knowledge on treating individuals with disabilities, and inadequate facilities (P < 0.01). However, no significant differences were found between the two groups regarding lack of dental insurance or dental anxiety (P < 0.001). Conclusions: This study confirmed the presence of socioeconomic disparities in the utilization of dental services among individuals with intellectual and developmental disabilities (IDD), alongside multiple barriers to accessing care. The findings identified the most commonly perceived obstacles to oral health services among individuals with IDD, providing valuable insights for the development of targeted health policies aimed at improving access to dental care and reducing oral health inequalities in this vulnerable population.  **Page # 2 & 3** |
| Introduction | | |
| Background/rationale | 2 | Explain the scientific background and rationale for the investigation being reported  Background: Oral health is an important component of overall well-being, yet individuals with intellectual and developmental disabilities (IDD) often experience significant challenges in accessing appropriate dental care. In Jordan, limited research has addressed these disparities, highlighting the need for a focused investigation  **Page # 4&5** |
| Objectives | 3 | State specific objectives, including any prespecified hypotheses  This study aimed to assess the utilization of dental services among individuals with IDD in Jordan and to identify key barriers affecting their access to dental care, compared to individuals without IDD  **Page # 5** |
| Methods | | |
| Study design | 4 | Present key elements of study design early in the paper  A cross-sectional survey was carried out to identify barriers to accessing oral healthcare for individuals with intellectual and developmental disabilities (IDD) in Jordan.A self-designed Arabic questionnaire was developed to ensure cultural and linguistic relevance for participants in Jordan. Content validity was assessed by two expert panels, achieving an average congruency percentage (ACP) of 92%, indicating a high level of agreement regarding item relevance and appropriateness. Reliability was evaluated using test–retest administration on 10 caregivers of individuals with IDD, with Cronbach’s alpha coefficient of 0.75, demonstrating acceptable internal consistency. A pilot study with 10 IDD’s caregivers (not included in the final sample) was also conducted to assess clarity, content, and format, and feedback was incorporated into the final version. The final questionnaire included three sections: 1.Demographic information (5 items) of participants and caregivers.2.Barriers to dental care (13 items), adapted and modified from the literature .3.Dental service utilization (5 items).  **Page # 5 &6** |
| Setting | 5 | Describe the setting, locations, and relevant dates, including periods of recruitment, exposure, follow-up, and data collection. A list of intellectual and developmental disability (IDD) centers was obtained from the Ministry of Social Development. A convenience sampling method was employed to recruit participants from centers located in the northern, southern, and central regions of Jordan. The selection of these centers was based on the willingness of special-care center managers to distribute the questionnaire to potential participants. Questionnaires were collected within one to three weeks of distribution. For non-respondents, a reminder note was sent after three weeks to encourage participation. Overall, data collection was conducted over a six-month period.  **Page # 6&7** |
| Participants | 6 | (*a*) Give the eligibility criteria, and the sources and methods of selection of participants  the eligibility criteria people with developmental delays  Centers listed in the Ministry of Social Development that agreed to participate distributed the paper-format questionnaire to parents/caregivers, who completed it on behalf of individuals with IDD. A cover letter explained the purpose of the study, emphasized voluntary participation, and assured confidentiality. For the comparison group, individuals without IDD were recruited from the same geographic areas using convenience sampling in schools, malls, and parks.  **Page # 6 &7** |
| Variables | 7 | Clearly define all outcomes, exposures, predictors, potential confounders, and effect modifiers. Give diagnostic criteria, if applicable  The study outcome are the barriers to accessing oral health care among individuals with IDD in Jordan, compared with their peers without IDD  Under the results section  **Page # 7 & 8** |
| Data sources/ measurement | 8* | For each variable of interest, give sources of data and details of methods of assessment (measurement). Describe comparability of assessment methods if there is more than one group  For IDD group: Descriptive statistics were calculated for continuous (mean, SD) and categorical variables (frequency, percentage). Associations were tested using Pearson’s Chi-square, with significance set at p < 0.05  For without IDD group: Descriptive statistics were calculated for continuous (mean, SD) and categorical variables (frequency, percentage). Associations were tested using Pearson’s Chi-square, with significance set at p < 0.05  **Page # 7 & 8** |
| Bias | 9 | Describe any efforts to address potential sources of bias  To minimize potential sources of bias, content validity was established through expert review, and a pilot study was conducted with 10 caregivers of individuals with intellectual and developmental disabilities (IDD) to refine the study instruments and procedures. The severity of disability for participants with IDD was obtained from official records maintained by center managers. These records were based on professional diagnostic evaluations documented in the participants’ files, thereby avoiding reliance on self-reported information. For the comparison group (individuals without IDD), participants were recruited from the same geographic regions using convenience sampling at locations such as schools, malls, and parks. This approach aimed to ensure demographic comparability between groups while maintaining practical feasibility.  **Page # 6** |
| Study size | 10 | Explain how the study size was arrived at  Sample size was calculated using G*Power software, based on Alshatrat et al. (2024) [28], with 80% power and a 5% margin of error. The minimum required sample size was 139 participants per group. A total of 200 questionnaires were distributed in each group. Of these, 168 were returned by caregivers of individuals with IDD and 149 by individuals without IDD. Return of a completed questionnaire was taken as implied consent.  **Page # 7** |
| Quantitative variables | 11 | Explain how quantitative variables were handled in the analyses. If applicable, describe which groupings were chosen and why  Data were analyzed using IBM SPSS (IBM-SPSS, Armonk, NY) with the assistance of a biostatistician. Descriptive statistics were calculated for continuous (mean, SD) and categorical variables (frequency, percentage). Associations were tested using Pearson’s Chi-square, with significance set at p < 0.05.  **Page # 7** |
| Statistical methods | 12 | (*a*) Describe all statistical methods, including those used to control for confounding |
|  |  | (*b*) Describe any methods used to examine subgroups and interactions |
|  |  | (*c*) Explain how missing data were addressed |
|  |  | (*d*) If applicable, describe analytical methods taking account of sampling strategy |
|  |  | (*e*) Describe any sensitivity analyses |
| Results | | |
| Participants | 13* | Report numbers of individuals at each stage of study—eg numbers potentially eligible, examined for eligibility, confirmed eligible, included in the study, completing follow-up, and analysed  A total of 200 questionnaires were distributed in each group. Of these, 168 were returned by caregivers of individuals with IDD and 149 by individuals without IDD. The response rate for people with developmental delays and people without developmental delays was 79% out of 200 eligible respondents for each group  **Page # 7 & 8** |
|  |  | Give reasons for non-participation at each stage  The primary reason for non-participation at all stages of the study was individuals’ unwillingness to participate. This included both caregivers of individuals with IDD and members of the comparison group. No participants were excluded due to ineligibility, and no dropouts occurred after consent was obtained. |
|  |  | (c) Consider use of a flow diagram |
| Descriptive data | 14* | Give characteristics of study participants (eg demographic, clinical, social) and information on exposures and potential confounders  Participants' ages ranged from 7 to 75 (mean age: 23 years) in the individual without IDD group and from 8 to 59 (mean age: 30 years) for those with IDD. Between the IDD group (61%: 39%) and the individual without IDD group (67%: 32%), the study population's male to female ratio was comparable. Moreover, 90% of people in both groups were single, and there is a significant difference in the average educational level between the two groups due to the individual without IDD group’s higher educational level (P < 0.05). Other demographic details are shown in Table 1, such as the location of developmental delay centers, the level of disability among participants with IDD, insurance coverage, and family income.  **Table 1** |
|  |  | (b) Indicate number of participants with missing data for each variable of interest: N/A |
| Outcome data | 15* | Report numbers of outcome events or summary measures  Using chi-square test, Table 2 presents the typical barriers that people face while trying to access and use dental services. There was a significant difference in the likelihood of reporting most barriers, with those with IDD significantly more likely to report them than individual without IDD (P value < 0.01), including lengthy wait times, high costs, the inconvenience of dental clinic hours, embarrassment, a lack of specialized dental staff, a lack of knowledge about how to treat people with disabilities, and inadequate facilities. However, there was no signiﬁcant difference between the two groups when it came to not having any insurance and dental anxiety (P value > 0.05).  **Table 2** |
| Main results | 16 | (*a*) Give unadjusted estimates and, if applicable, confounder-adjusted estimates and their precision (eg, 95% confidence interval). Make clear which confounders were adjusted for and why they were included  NA |
|  |  | (*b*) Report category boundaries when continuous variables were categorized  NA |
|  |  | (*c*) If relevant, consider translating estimates of relative risk into absolute risk for a meaningful time period  NA |
| Other analyses | 17 | Report other analyses done—eg analyses of subgroups and interactions, and sensitivity analyses  NA |
| Discussion | | |
| Key results | 18 | Summarise key results with reference to study objectives  Findings indicated that toothache was the most common reason for dental visits in both groups, though individuals with IDD were significantly more likely to visit the dentist only when in pain. This suggests a pattern of symptomatic rather than preventive care, often delaying visits until oral conditions progressed to urgent severe dental problems. Compliance with follow-up appointments was also higher among individuals with IDD, likely reflecting the need for continued care following urgent treatment. In both groups, routine dental checkups were uncommon, despite strong evidence linking regular checkups to better oral and general health outcomes.Caregivers of individuals with IDD reported multiple barriers, including geographical, physical, behavioral, cultural, human resource, and financial challenges.Such barriers impede timely, preventive care and exacerbate health disparities.Insurance coverage was not perceived as a barrier in either group, suggesting that governmental health services provide a baseline level of access. However, financial inadequacy remained a major barrier for caregivers of individuals with IDD, most of whom were from low-income families. This indicates that although insurance is available, it is insufficient to cover the specialized dental care often required. Similar findings have been reported elsewhere, where socioeconomic disparities were linked to reduced dental service utilization and poorer oral health outcomes  Fear of dental treatment was reported by about half of participants in both groups, with no significant difference. This aligns with evidence that dental anxiety affects a substantial proportion of the general population. In individuals with IDD, anxiety may be influenced by age and severity of disability, which could explain the lack of group differences observed in this study. Emerging behavior guidance techniques, such as audio or VR distraction, may be beneficial but remain underexplored in adults with IDD.  Physical and logistical barriers were also prominent. Caregivers highlighted difficulties with wheelchair access, inadequate facilities, long waiting times, lack of parking, and inconvenient clinic hours. Such obstacles are consistent with previous reports and underscore the importance of designing inclusive, accessible dental facilities.  Human resource limitations were noted, particularly the lack of dentists trained to manage individuals with IDD. Caregivers expressed concern about both knowledge gaps and the limited availability of specialists, consistent with earlier findings [56]. Cultural barriers, including embarrassment and stigma, also hindered access. These findings point to the need for increased provider training, caregiver support, and public awareness to foster inclusivity and reduce stigma.  Although the literature on barriers to dental services among individuals with IDD is limited, the current findings align with prior research indicating that cost, accessibility, and limited specialized services are key obstacles.Interventions to address these barriers may include tailored oral health education, improved access pathways, caregiver empowerment, cultural inclusivity initiatives, and policies to ensure affordable specialized care.  **Page # 8-10** |
| Limitations | 19 | Discuss limitations of the study, taking into account sources of potential bias or imprecision. Discuss both direction and magnitude of any potential bias  This study has several limitations. The findings reflect caregiver perspectives rather than those of individuals with IDD, and only institutionalized individuals were included, which may not represent the broader population. There was also a mismatch in age and family income distributions between groups. Although a reliable questionnaire was used, direct interviews could have provided richer data.  **Page # 11** |
| Interpretation | 20 | Give a cautious overall interpretation of results considering objectives, limitations, multiplicity of analyses, results from similar studies, and other relevant evidence  To the best of our knowledge, this is the first study to examine barriers to dental services among individuals with (IDD). As such, it provides valuable insights into a disadvantaged population and can serve as a baseline for larger, more comprehensive studies investigating oral health needs and barriers to care.  **Page # 11** |
| Generalisability | 21 | Discuss the generalisability (external validity) of the study results  only institutionalized individuals were included, which may not represent the broader population  **Page # 11** |
| Other information | | |
| Funding | 22 | Give the source of funding and the role of the funders for the present study and, if applicable, for the original study on which the present article is based  This research was funded by Jordan University of Science and Technology Research Grant No: 20170032  **Page # 11** |

*Give information separately for exposed and unexposed groups.

**Note:** An Explanation and Elaboration article discusses each checklist item and gives methodological background and published examples of transparent reporting. The STROBE checklist is best used in conjunction with this article (freely available on the Web sites of PLoS Medicine at http://www.plosmedicine.org/, Annals of Internal Medicine at http://www.annals.org/, and Epidemiology at http://www.epidem.com/). Information on the STROBE Initiative is available at www.strobe-statement.org.
