# Supplementary material for: Comparative assessment of dental care services utilization and barriers among individuals with and without intellectual and developmental disabilities in Jordan
Source: PeerJ. 2026 Jun 18;14:e21447. doi: 10.7717/peerj.21447 (PMC13283370; doi:10.7717/peerj.21447)
Supplement: Supplemental Information 3 [file peerj-14-21447-s003.docx]

**Oral Health Knowledge, Behaviors, and Dental Service Utilization among Persons with Disability Questionnaire**

**DIRECTIONS:** Please answer each question by checking (x) the answer that BEST reflects your knowledge or by writing in a short response.

**SECTION1: Demographics**

**Questions for Person with Disability**

Date of birth of a person with disability: --------------------

Gender of a person with disability: Male Female

Monthly Income:

< 250 250-500 >500-1000 > 1000 Does Not Work

Educational Level (check the highest):

Elementary Middle School High School College University Graduate

Marital Status

Married Unmarried Others

Health Insurance yes No

**SECTION 2:** Oral Health Knowledge

1. Dental plaque is formed by colonizing bacteria trying to attach themselves to the tooth’s surface.

- Yes
- No
- Do not know

1. Dental caries mainly caused by the bacteria

- Yes
- No
- Do not know

1. Having sugars can lead to the dental caries or do sweet affect dental health

- Yes
- No
- Do not know

1. Having soft drinks affect dental health:

- Yes
- No
- Do not know

1. Is there any relationship between oral health and overall health?

- Yes
- No
- Do not know

1. It is normal for your ٍ:

- Yes
- No
- Do not know

1. It is normal for your gum to be red

- Yes
- No
- Do not know

1. It is normal for your gum to be swelling

- Yes
- No
- Do not know

1. Brushing teeth on regular basis protect your teeth

- Yes
- No
- Do not know

1. You need to visit a dentist only when you have a toothache

- Yes
- No
- Do not know

1. You need a hard toothbrush to clean your teeth

- Yes
- No
- Do not know

1. Dental floss is necessary to keep your teeth clean

- Yes
- No
- Do not know

1. Missing tooth/teeth could be replaced

- Yes
- No
- Do not know

1. Caries may lead to loos natural tooth/teeth

- Yes
- No
- Do not know

1. You need to visit a dentist to replace any missing tooth/teeth

- Yes
- No
- Do not know

**SECTION 3:** Oral health behavior

1. Frequency of tooth brushing [how often do you brush your teeth]
   - brushing at least twice a day
   - brushing once a day
   - seldom or no brush
2. Time spend during brushing
   - less than a minute
   - more than a minute but less than 2 minutes
   - more than 2 minutes
3. How often do you floss your teeth?
   - once a day or more
   - none
4. How often do use mouthrinse
   - once a day or more
   - none
5. Use of fluoridated toothpaste
   - fluoridated
   - no- fluoridated
   - have no idea
6. Frequency of eating sweets
   - two times or more per day
   - once a day
   - none
7. Frequency of drinking soda
   - one can
   - 2 cans per day
   - 3 cans per day
   - 4 and more

**SECTION 4: Dental Service use**

- 1. Time since last visit to the dentist
     - Less than a year
     - 1-2 years ago
     - 3 or more years ago
  2. What was the main reason you last visited the dentist?
- A checkup visits
- Follow-up for a known reason (maintenance)
- Emergency visit
- Toothache
- Other

**Barriers to Access to Dental Care**

What were the reasons that disabled person could not get the dental care [he/she] needed? Check **ALL** that apply

- Could not afford the cost
- Dental office is too far away
- Dental office is not open at convenient times
- Dental office has no or difficult access for wheelchair
- Dental office has inaccessible parking areas
- Dental office has small space, narrow doorways, internal steps which make access to dental office is difficult
- Dental office has inadequate facilities to provide dental care, such as: special chair, disabled toilet access, sedation
- Dentist lack of knowledge of how to treat people with disability
- Dental office has a general dentist not a specialist
- Long waiting time
- Fear of dental work
- No Insurance coverage/dental coverage
- Embarrassment or any psychological barriers.

**THANK YOU**
