# Supplementary material for: Comparative assessment of dental care services utilization and barriers among individuals with and without intellectual and developmental disabilities in Jordan
Source: PeerJ. 2026 Jun 18;14:e21447. doi: 10.7717/peerj.21447 (PMC13283370; doi:10.7717/peerj.21447)
Supplement: Supplemental Information 4 [file peerj-14-21447-s004.docx]

المعرفة و السلوكيات تجاه الصحة الفموية واستخدام خدمات الاسنان

إرشادات : أرجو الإجابة على الأسئلة التالية بوضعx دائرة حول الاجابة الصحيحة و إكمال الفراغ .

**القسم الاول : معلومات عامة**

| **تاريخ الميلاد**:……………………………. | | | | | | | | | |  |
| --- | --- | --- | --- | --- | --- | --- | --- | --- | --- | --- |
| **الجنس**: | | ذكر | | | | انثى | | | |  |
| **الدخل الشهري للعائلة**: | | | | | | | | | |  |
|  | لا يعمل اقل من 250 دينار | | | | من 250-500 دينار | أكثرمن 500-1000 دينار | | اكثر من 1000 دينار | |  |
| **التحصيل العلمي.** | | | | | | | | | |  |
| المرحلة الابتدائية | | | المرحلة الإعدادية | | | المرحلة الثانوية | كلية | جامعة | دراسات عليا | |
| **هل لديك تأمين صحي** : | | | | نعم | | لا | | | |  |

ال**قسم الثاني: المعرفة بخصوص الصحة الفموية**

**ضع دائرة حول الخيار المناسب :**

| 1. اللويحة الجرثومية تتكون من تجمعات بكتيرية تحاول الالتصاق على سطح السن | | | | |
| --- | --- | --- | --- | --- |
|  | نعم | | لا | لا أدري |
| 2. البكتيريا هي السبب الرئيسي لتسوس الأسنان | | | | |
|  | نعم | | لا | لا أدري |
| 3. تناول الحلويات يؤدي إلى تسوس الأسنان | | | | |
|  | نعم | | لا | لا أدري |
| 4. تناول المشروبات الغازية مثل البيبسي يؤثر في الصحة الفموية | | | | |
|  | نعم | | لا | لا أدري |
| 5. هل هناك علاقة بين الصحة الفموية وصحة الجسم؟ | | | | |
|  | نعم | | لا | لا أدري |
| 6. من الطبيعي ان تنزف لثتك خلال تفريش اسنانك | | | | |
|  | نعم | | لا | لا أدري |
| 7. من الطبيعي ان تكون لثتك حمراء | |  |  |  |
|  | نعم | | لا | لا أدري |
| 8. من الطبيعي ان تكون لثتك متورمة | | | | |
|  | نعم | | لا | لا أدري |
| 9. تفريش اسنانك بشكل منتظم يحمي اسنانك | | | | |
|  | نعم | | لا | لا أدري |
| 10. تحتاج إلى زيارة طبيب الأسنان فقط عندما يكون هناك الــــم | | | | |
|  | نعم | | لا | لا أدري |
| 11. تحتاج إلى استخدام فرشاة اسنان قاسية لتنظيف اسنانك | | | | |
|  | نعم | | لا | لا أدري |
| 12. الخيط السني مهم للمحافظة على نظافة اسنانك | | | | |
|  | نعم | | لا | لا أدري |
| 13. الاسنان المخلوعة او المفقودة ممكن تعويضها اواستبدالها | | | | |
|  | نعم | | لا | لا أدري |
| 14. تسوس الأسنان قد يؤدي فقدان الأسنان الطبيعية | | | | |
|  | نعم | | لا | لا أدري |
| 15. تحتاج الى زيارة طبيب الأسنان لتعويض السن اوالاسنان المفقودة | | | | |
|  | نعم | | لا | لا أدري |

ال**قسم الثالث: التصرفات الخاصة بالصحة الفموية**

**ضع دائرة حول الخيار المناسب :**

| 1. كم عدد المرات التي تفرشي فيها اسنانك ؟ | | | | | | |
| --- | --- | --- | --- | --- | --- | --- |
|  | مرة واحدة في اليوم | | على الأقل مرتين في اليوم | نادرا او لا افرشي اطلاقا | | |
| 2. كم الوقت الذي تأخذه في تفريش اسنانك؟ | | | | | | |
|  | أقل من دقيقة | | أكثر من دقيقة ولكن اقل من دقيقتين | أكثر من دقيقتين | | |
| 3. كم عدد المرات التي تستخدم فيها الخيط السني؟ | | | | | | |
|  | مرة واحدة او أكثر في اليوم | | ولا مرة |  | | |
| 4. كم عدد المرات التي تستخدم فيها المضامض الفموية ؟ | | | | | | |
|  | مرة واحدة في اليوم او اكثر | | ولا مرة |  | | |
| 5. هل معجون الاسنان خاص بك ؟ | | | | | | |
|  | يحتوي على الفلورايد | | لا يحتوي على الفلورايد | لا أدري | | |
| 6. كم عدد المرات التي تتناول فيها الحلويات؟ | | | | | | |
|  | مرتين او أكثر في اليوم | | مرة واحدة في اليوم | احيانا | ولا مرة | |
| 7. كم عدد المرات التي تتناول فيها المشروبات الغازية مثل البيبسي ؟ | | | | | | |
| ولا مرة | | علبة واحدة في اليوم | علبتان في اليوم | ثلاث علب او اكثر في اليوم | | احيانا |

**القسم الرابع : إستخدام الخدمات السنية**

ضع دائرة حول الخيار المناسب :

| 1. اخر زيارة لطبيب الاسنان كانت | | | | | | |
| --- | --- | --- | --- | --- | --- | --- |
|  | | | اقل من سنة | من سنة الى سنتين | ثلاث سنوات او أكثر | |
| 2. ماذا كان السبب لهذه الزيارة ؟ | | | | | | |
| زياره روتينية | | | متابعة | طارئة | الم في الاسنان | اخرى |
| 3. ما هي الأسباب التي تمنعك وتعيقك من العناية بالصحة الفموية التي تحتاجها ؟ | | | | | | |
| **ضـــــع إشـــــارة علـــــى كل ما ينطبق** | | | | | | |
| 1. |  | لا تستطيع تحمل التكاليف | | | | |
| 2. |  | عيادة الأسنان بعيدة جدا | | | | |
| 3. |  | عيادة الأسنان تفتح في أوقات غير مناسبة لي | | | | |
| 4. |  | عيادة الأسنان لاتحتوي على مصفات خاصة للسيارات | | | | |
| 5. |  | عيادة الأسنان صغيرة، تحتوي على ممرات ضيقة، ودرج داخلي | | | | |
| 6. |  | عيادة الأسنان لا تحتوي بشكل كافي على أجهزة ومعدات ضرورية لتقديم العناية السنية | | | | |
| 7. |  | عيادة الأسنان يوجد فيها طبيب أسنان عام وليس اختصاص | | | | |
| 8. |  | وقت الانتظار طويل | | | | |
| 9. |  | الخوف من العمل السني | | | | |
| 10. |  | لا يوجد تامين صحي اوتامين للاسنان | | | | |
| 11. |  | الشعور بالاحراج والضغط النفسي | | | | |
